# User Engagement With a Mobile Health App for People Living With HIV: Observational Study Based on an Engagement Evaluation Framework

**DOI:** 10.2196/78810

**Published:** 2025-09-09

**Authors:** Gwang Suk Kim, Seoyoung Baek, Layoung Kim, Sooyoung Kwon

**Affiliations:** 1 Mo-Im Kim Nursing Research Institute, College of Nursing, Yonsei University, Seoul, Republic of Korea; 2 Korea Armed Forces Nursing Academy, 90, Jaun-ro, Yuseong-gu, Daejeon, 34059, Republic of Korea, 82 10-4714-2111; 3 Department of Nursing, The University of Suwon, Hwaseong, Republic of Korea; 4 College of Nursing, Yonsei University, Seoul, Republic of Korea

**Keywords:** HIV, mobile apps, patient engagement, mobile health, mHealth, smartphone, digital health

## Abstract

**Background:**

Mobile health (mHealth) interventions can be effective for people living with HIV, who are sensitive to privacy breach risks. Understanding the perceived experiences of intervention participants can provide comprehensive insights into potential users and predict intervention effectiveness. Thus, it is necessary to plan engagement measurement and consider ways to enhance engagement during the app development phase.

**Objective:**

This study aims to evaluate engagement with a mHealth app using the engagement index and time and to examine differences in engagement according to participant characteristics.

**Methods:**

This observational study, conducted from March 27 to August 31, 2024, was based on an engagement evaluation framework. A total of 261 people living with HIV across 13 medical institutions and 3 related organizations in South Korea engaged in an app-based intervention for 4 weeks. Self-reported surveys were conducted before and after the app usage period, and usage data were collected during the intervention to assess user engagement. User engagement was evaluated using 2 measures: engagement index and engagement time. The engagement index represents a standardized percentage derived from 5 subindices: click depth, loyalty, recency, feedback, and interaction.

**Results:**

The median engagement index and time were 67.6% (IQR 59.7‐74.1) and 27.2 minutes (IQR 4.9‐81.4), respectively, with a statistically significant positive correlation between the two (*ρ*=0.589; *P*<.001). The engagement index was higher among those with self-help group participation (*U*=6014.0, *z*=−2.49; *P*=.01). There were no differences in engagement index and time according to other personal characteristics.

**Conclusions:**

When developing mHealth apps for people living with HIV, it is essential to track both objective indicators such as login data and subjective indicators such as patient experience for comprehensive intervention evaluation. Based on this study’s results, high engagement suggests that apps should prioritize user needs through rewards, privacy protection, tailored information, and esthetic features. While the app in this study demonstrated inclusive usability, targeted support strategies may benefit users without self-help group participation.

## Introduction

Digital health is recognized as a critical catalyst for advancing universal health coverage, and the use of digital health is a globally recommended channel of practice for strengthening health care services and allowing practitioners to significantly improve health outcomes [1]. Among people living with HIV, mobile health (mHealth) interventions have demonstrated significant improvements in medication timing compliance, highlighting their therapeutic potential [2]. mHealth for people living with HIV has been continuously developed to increase engagement in health care while being less time- and cost-intensive than other options [3]. Recent studies have demonstrated that mHealth interventions can significantly improve operational efficiency in HIV services, with potential reductions in clinic visits by up to 14% and substantial cost savings while maintaining quality care [4]. Given that electronic health care programs demonstrate excellence in delivering accessible care and enhancing patient engagement [5], there is increasing potential for health care provider–led mHealth initiatives targeting people living with HIV. This approach could be an effective strategy to help individuals overcome personal challenges, such as stigma and discrimination, that impede their access to health care services. However, studies examining mHealth apps for people living with HIV remain limited in the literature.

When users demonstrate strong engagement and report positive experiences such as enjoyment, the effectiveness of digital behavioral change interventions is expected to increase [6]. Higher levels of engagement also show a significant correlation with improved postintervention mental health outcomes [7]. In an app intervention study that applied cognitive behavioral stress management courses and physical activity promotion for people living with HIV with high levels of depression, the group with higher engagement showed significantly greater reductions in depressive symptoms and perceived stress, along with improved quality of life [8]. This suggests that implementing mHealth programs with enhanced engagement among people living with HIV could be an effective approach to improving health-related outcomes in this population. Thus, engagement assessment should be considered during app development and implementation.

While various studies on mHealth development and engagement measurement have been reported, there is no agreed-upon definition of user engagement, directly impeding interdisciplinary communication about this topic [9]. App engagement may be defined as the extent of usage (eg, amount, frequency, duration, and depth) and a subjective experience characterized by attention, interest, and affect [6]. Most previous studies on mHealth interventions for people with chronic diseases have measured engagement using either a single metric or a combination of 2 metrics, with 54.8% using login data such as login counts and 26.4% measuring direct data entry into apps [9]. Puig et al [10] evaluated engagement in people living with HIV aged 60 years and older using metrics such as frequency of use and session duration, finding that 73.8% of the participants used their app for a mean of 23.7 days over 48 weeks. This single-metric approach, while providing valuable usage insights, lacks the comprehensive assessment needed to understand the multidimensional nature of user engagement in mHealth interventions. Beyond physical participation metrics, understanding users’ psychological states and perceived experiences can provide a more comprehensive understanding of the target audience’s behaviors [11]. The engagement index can be a useful tool for analyzing the usability of mHealth apps as it measures both objective and subjective user experiences [12]. While the total time spent on an app is not a component of the engagement index, it can serve as a complementary metric by providing insight into the duration of app usage beyond mere frequency of access.

Upon reviewing measurement tools for user engagement [9,13], the engagement index and time were found to be comprehensive elements capable of measuring app feature utilization. The login data used to calculate the engagement index offer the advantage of more accurately reflecting user engagement than do subjective participant reports, being free from social desirability and recall bias [9]. Therefore, the aim in this study is to examine and compare the engagement index and engagement time by defining them as elements of an engagement evaluation framework, demonstrating how these elements can be used in analyzing mHealth app engagement. An additional aim is to investigate whether app engagement differs according to participant characteristics.

## Methods

### Design

This study employed an observational design. It was implemented based on an engagement evaluation framework to assess the efficacy of mobile care navigation in HIV treatment and health care management.

### Study Overview

The research team developed a mobile app (“Excellent Self-Supervised HIV Care [ESSC]”) for people living with HIV to improve their self-health care management [14]. The ESSC app was designed to facilitate integrated HIV care by combining users’ needs, therapeutic goals, and medical expertise [14]. The app consists of 5 main functions: information, medication management, mental health, question and answer (Q&A) with nurses, and My Page. It provides verified disease- and health-related information content and includes self-health recording features that support medication adherence and emotional health.

Version 2 of the ESSC app is an improved version over the original based on pilot study participants’ feedback [15]. To make it more practical for commercialization compared with its initial specifications, specific functions were carefully selected to clarify the app’s operational purpose. Through the app, the research team aimed to meet user needs and enhance interaction via periodic updates of the latest information, active user commenting features, and administrator messaging capabilities. To encourage engagement and communication between researchers and participants as well as among participants, a mileage-based incentive system was implemented. Users received mileage points based on their activity levels, with additional incentives provided according to accumulated mileage amounts. Specific activities were rewarded as follows: medication recording, mood diary entries, sexual health recording, rating information content, and commenting each earned 100 points; public Q&A posts earned 2000 points; and private Q&A posts earned 1000 points. A single-arm pre-post study was conducted using version 2 of the ESSC app to evaluate user data and use of the mobile app.

This study was performed from March 27 to August 31, 2024. Participants used ESSC version 2 for 4 weeks after enrollment and completed self-reported surveys before and after the 4-week app usage period. Participants who wished to continue using the app were free to do so until the end of the study. Usage data collected throughout the app usage period were used.

### Participants

The inclusion criteria were people living with HIV aged 19 years or older who used smartphones, could read and respond to questionnaires via smartphones without impairment, and could download and use mobile apps without difficulty. People living with HIV who had participated in the pilot study were excluded. A total of 268 app users who consented, completed the pre-test, and were registered on the mobile app participated in the study. After excluding 7 participants who did not complete the post-intervention survey, data of 261 people living with HIV were ultimately included in the analysis.

### Procedure and Data Collection

Participants were recruited using probabilistic convenience and snowball sampling. The research team collaborated with 13 medical institutions and 3 HIV-related organizations and community networks spread across many cities in South Korea. In clinical settings, physicians and consulting nurses distributed recruitment cards to people living with HIV during routine outpatient visits to infectious disease departments. Web-based recruitment was conducted through posts on HIV-specific community platforms and networks that are exclusively accessible to people living with HIV to ensure participant confidentiality and targeted outreach. Considering the sensitive nature of HIV status disclosure, information cards were designed in a pocket-sized format and carefully distributed to avoid public exposure. The information cards and online posts included the research objectives and participation procedures, as well as a QR code to access the web-based baseline survey. Participants could access the web-based survey only after reviewing and accepting the informed consent statement on the website. Following the baseline survey, the research team contacted eligible participants using the collected contact information to provide detailed information about the mobile app program and study procedures. Participants received text-messaging instructions for app installation via the App Store or Google Play. Login access was granted only after research team verification.

The mobile app health management program was implemented considering the characteristics of people living with HIV, who experience difficulties in communication and sharing information with unfamiliar health care providers. The researchers and participants did not have direct face-to-face contact. Participants were able to review the necessary information for informed decision-making and confirm their consent on the web. After voluntarily deciding to participate in the study, people living with HIV were directed to complete 2 web-based surveys (at baseline and at the 4-week intervention end point). The baseline survey included questions about participants’ general characteristics, such as age, marital status, living status, educational level, employment, perceived economic status, HIV care stage, and self-help group participation. The 4-week survey contained items from the Health Information Technology Usability Evaluation Scale (Health-ITUES) [16]. During the intervention period, app usage logs including session information and event occurrence data were collected.

### Variables and Measures

#### Engagement Index

##### Overview

The Web Analytics Demystified Visitor Engagement Index [12] was adapted to develop a measure of engagement for ESSC app users. The original index comprised 7 subindices: click depth, loyalty, recency, interaction, feedback, brand, and duration. Five of these 7 subindices were used in this study. Each subindex captures a different aspect of engagement: click depth (content exploration), loyalty (return visits), recency (recent activity), feedback (satisfaction), and interaction (active participation) (Table 1) [12]. The engagement index represents the standardized percentage of the sum of 5 subindices. Each subindex is first normalized to a 0‐100 scale, after which the 5 scores are averaged to create the overall engagement index ranging from 0 to 100. The index was customized to suit the ESSC intervention and was meant to rank participants.

**Table 1.** Calculation of subindices for the engagement index measuring mHealth app usage among people living with HIV.

Sub-index	Formula
Click depth	Sessions having at least 4 pages viewed/all sessions
Loyalty	1 − (1/number of sessions accessed during the intervention)
Recency	1/average number of days between visits for each period
Feedback	Score of Health-ITUES^a^/total score of Health-ITUES (100)
Interaction	[(Number of comments/maximum number of comments) + (number of questions/maximum number of questions) + (number of ratings given/maximum number of ratings given)]/3

a Health-ITUES: Health Information Technology Usability Evaluation Scale.

##### Click-Depth Index

The click-depth index measures user attention by analyzing how deep users navigate within a website during each session. While traditional metrics rely on page views per session, this index reduces statistical noise by only including sessions where users exceed a specified click-depth threshold, thereby filtering out brief visits where users quickly leave after viewing minimal pages. In this study, sessions with 4 or more page views were used as the threshold to identify meaningful engagement. Based on our exploration of the log data, meaningful health management behaviors required a minimum of 4-page views, excluding sessions where users simply accessed the app without substantial engagement. For example, a user who navigated from the home screen→medication input screen→medication date selection screen→completing medication input would generate 4-page views, meeting the meaningful engagement threshold.

##### Loyalty Index

The loyalty index functions as an indicator of user attention by tracking repeat website visits. This metric is based on the logical assumption that visitors who consistently return to an app are demonstrating some level of engagement with its content. The scoring system creates a spectrum where one-time visitors receive 0%, while highly active users who visit frequently during the measurement period earn scores approaching 100%. This gradual scale effectively captures the varying degrees of user engagement, suggesting that more frequent visits correlate with higher levels of attention and interest in the app. For instance, a user who visits the app 15 times during a 28-day period would receive a higher loyalty score than someone who visits only thrice.

##### Recency Index

The recency index evaluates how frequently a visitor has accessed the site in the recent past. The timing of a user’s last interaction is the strongest indicator of their future actions—the more recent their activity, the higher the probability that they will repeat it. In this study, the number of days between sessions was calculated to derive the recency index. For example, a user whose last session was 1 day ago receives a higher recency score than someone whose last session was 7 days ago.

##### Feedback Index

The feedback index stands as the fundamental qualitative measure used to determine visitor engagement levels. To thoroughly examine participants’ app usage experience, the Korean version of the Health-ITUES [16] was administered in the post-test. This instrument comprises 20 items rated on a 5-point Likert scale, with total scores ranging from 20 to 100.

##### Interaction Index

**Figure 1.**
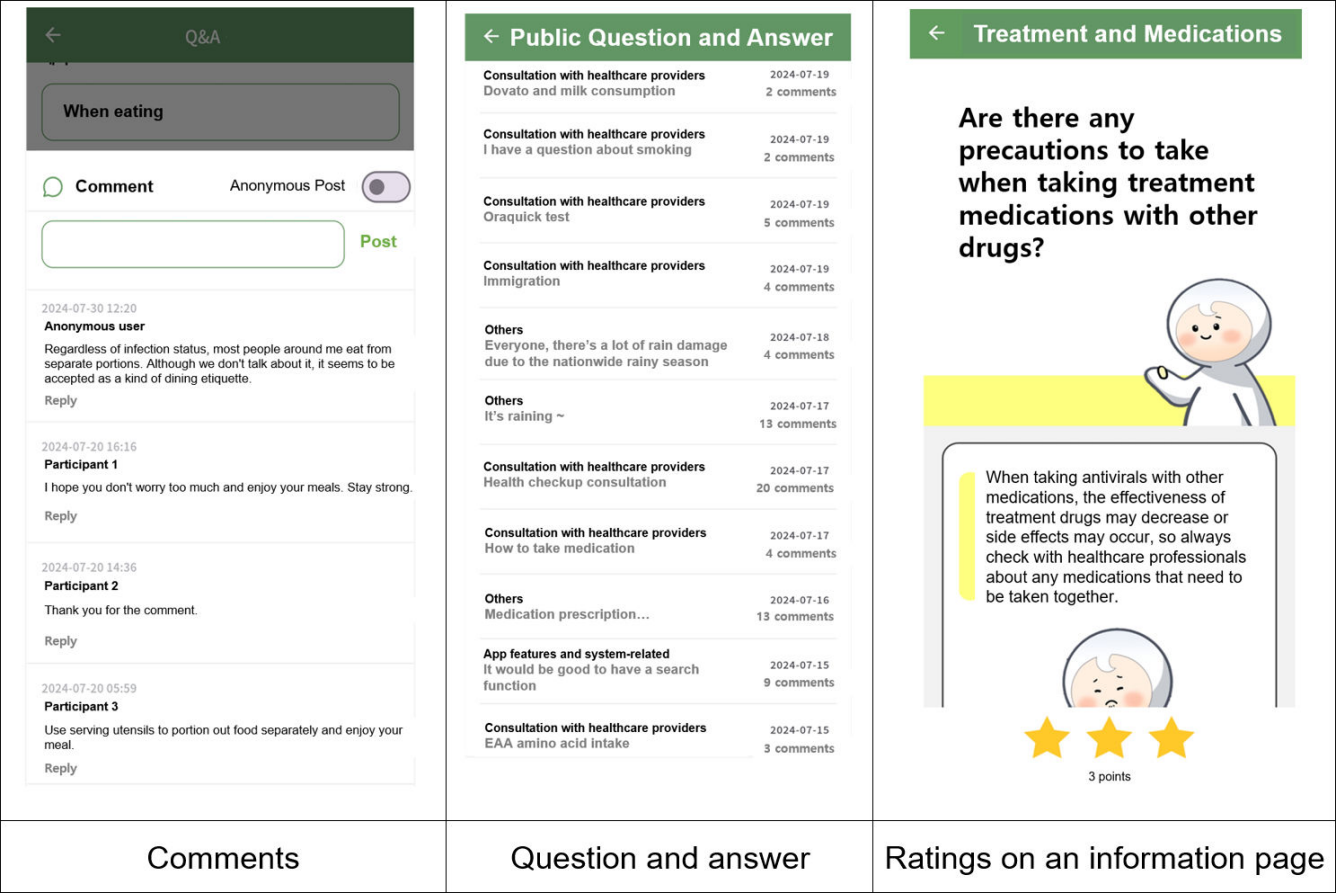
Screenshot of app interaction features (comments, question and answer, and information ratings) used for calculating interaction index.

The interaction index tracks visitor behavior by measuring specific user actions on the app. Among all the subindices in this study’s framework, it offers the greatest flexibility but requires organizations to invest significant effort in customizing it to their specific needs. The ESSC app used as an intervention in this study featured interactive elements such as public and private Q&A, comments, and content-rating systems, enabling interactions between researchers and participants as well as among participants themselves (Figure 1). The numbers of questions, comments, and ratings submitted by participants were used to calculate the interaction index.

### Engagement Time

Engagement time refers to the total cumulative duration (minutes) participants spent using the app during the 28-day intervention period. A system automatically collected tracking data whenever participants accessed the app, including time stamps for each user’s app visits and detailed records of when they entered and left specific content pages.

### Statistical Analysis

The engagement data automatically collected from the system were preprocessed using Python (version 3.9.10; Python Software Foundation) and subsequently analyzed with SPSS (version 29.0; IBM Corp). A descriptive statistical analysis was conducted on both the individual components and final score of the engagement index and engagement time.

Prior to statistical analysis, normality assumptions were examined using the Kolmogorov-Smirnov test, skewness values, and Q-Q plots. The Kolmogorov-Smirnov test results were significant for both engagement index (*P*<.001) and engagement time (*P*<.001), indicating nonnormal distributions. Additionally, the absolute skewness values exceeded 1 for both variables (engagement index: −1.63; engagement time: 3.62), and Q-Q plots showed substantial deviations from the 45° reference line. Based on these findings, nonparametric statistical methods were deemed appropriate for the analysis. Engagement index and time comparisons according to participant characteristics were conducted using the Mann-Whitney *U* test for 2-group comparisons and the Kruskal-Wallis test for multiple-group comparisons. Correlations among variables were assessed using Spearman rank correlation coefficient.

### Ethical Considerations

This study received ethical approval from the Institutional Review Board of Yonsei University Health System (number 4-2023-1609; January 30, 2024). People living with HIV voluntarily participated after reviewing information about the research purpose and procedures through information cards and web-based posts, and the survey could be initiated only after clicking the web link and providing informed consent. Informed consent included permission for secondary data analysis for academic research purposes. Phone numbers and email addresses were collected for participant recruitment, incentive provision, and mobile app intervention implementation. All analytical data were stored separately from personally identifiable information. Data collected for research purposes were encrypted and stored on the researcher’s password-protected computer to maintain data security. Personal information was collected only after participants reviewed the research information and provided consent. All collected data were used exclusively for research purposes and secondary data analysis for academic research. In cases of secondary data analysis, data may be accessed by relevant research team members as third parties, but all data are stored in a deidentified format with strict confidentiality maintained. People living with HIV received mobile gift cards (equivalent to 50,000 Korean won, approximately US $36) as appreciation for research participation. Additional mobile gift cards (up to 50,000 Korean won, approximately US $36) were provided based on accumulated mileage according to mobile app usage levels.

## Results

### Active User Trends Over Time

**Figure 2.**
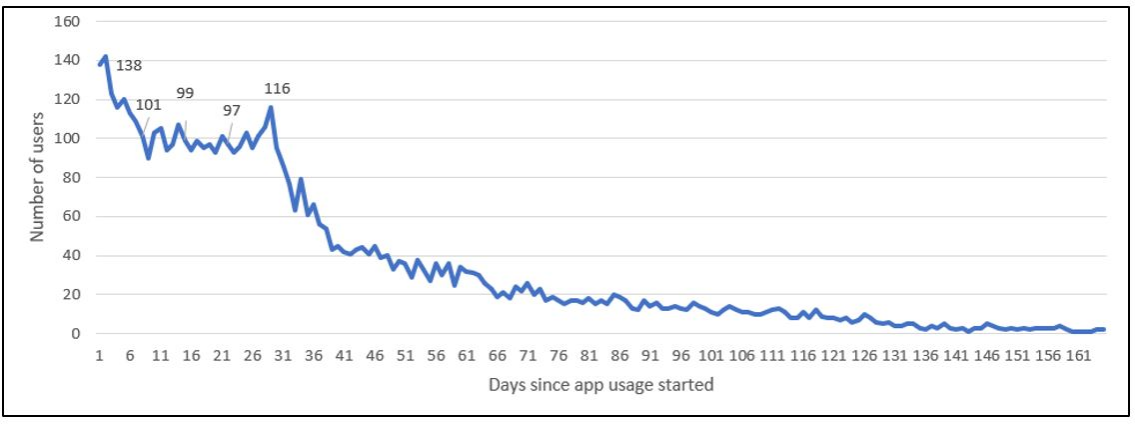
Number of app users by days since initial app usage among people living with HIV, March-August 2024 (N=261).

During the 28-day study period, the median number of sessions on the ESSC app was 28.0 (IQR 11.0‐47.0). Figure 2 demonstrates the number of ESSC app users over time from the beginning of app usage. On the first day of the study, 138 out of 261 participants used the app. After 1 week, the number decreased to 101 users, further reducing to 99 users in 2 weeks and 97 users in 3 weeks. By the fourth week, app users increased to 116.

### Engagement Index and Time

**Figure 3.**
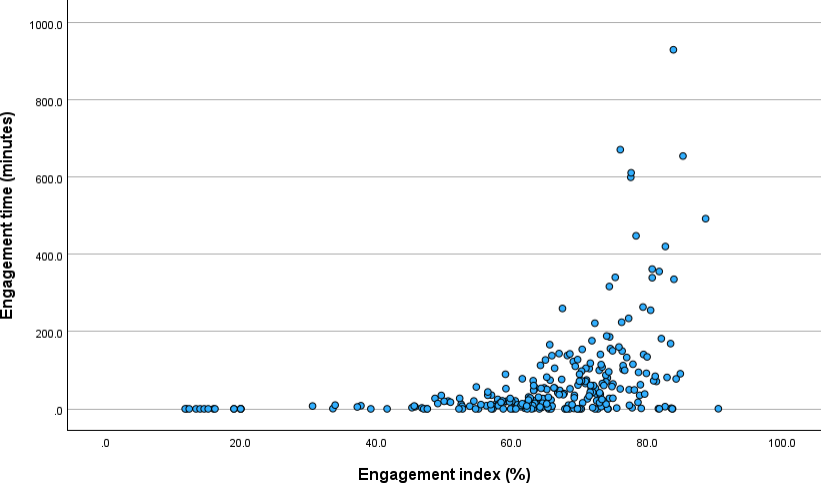
Correlation between engagement index and engagement time among people living with HIV using an mHealth app (N=261).

The median engagement index score was 67.6% (IQR 59.7‐74.1). Examining the 5 subindices, the medians were as follows—click-depth index: 84.2 (IQR 75.0‐92.3), loyalty index: 96.4 (IQR 90.9‐97.9), recency index: 62.5 (IQR 34.6‐88.5), interaction index: 5.3 (IQR 0.0‐32.0), and feedback index: 84.0 (IQR 74.0‐96.0). During the study period, the median engagement time was 27.2 (IQR 4.9‐81.4) minutes. A statistically significant positive correlation was found between the engagement index and engagement time (ρ=0.589; *P*<.001) (Figure 3).

### Engagement Index and Time According to Participant Characteristics

The engagement index and time according to participant characteristics are shown in Table 2. The median age was 41.0 (IQR 33.0‐53.0) years. Those with self-help group participation showed statistically higher median engagement index scores (70.1 out of 100) than those with no self-help group participation experience (65.8 out of 100) (*U*=6014.0, *z*=−2.49; *P*=.01).

**Table 2.** mHealth app engagement index and time according to participant characteristics among people living with HIV (N=261).

Variable	Values, n (%)	Engagement index			Engagement time		
		Median (IQR)	U/Z^a^, H^b^	*P* value	Median (IQR)	U/Z, H	*P* value
Age (years)			0.78	.86		4.43	.22
<29	43 (16.4)	67.7 (10.2)			26.0 (49.3)		
30‐39	83 (31.8)	68.6 (14.4)			27.3 (54.9)		
40‐49	61 (23.4)	66.2 (14.8)			54.6 (120.3)		
≥50	74 (28.4)	69.1 (18.4)			22.8 (108.3)		
Marital status			2124.0/−0.88	.38		2368.0/−0.13	.90
Having a spouse	20 (7.7)	64.7 (18.0)			28.8 (75.7)		
No spouse	241 (92.3)	67.8 (14.0)			27.2 (78.6)		
Living status			7983.5/−0.54	.59		8198.5/−0.18	.86
Living alone	142 (54.4)	68.2 (15.0)			27.3 (82.7)		
Living with others	119 (45.6)	67.1 (13.9)			27.3 (75.4)		
Educational level			7527.5/−1.35	.18		8114.5/−0.38	.70
≤High school	112 (42.9)	65.6 (17.2)			27.8 (86.8)		
≥College, university	149 (57.1)	69.2 (12.7)			26.7 (68.1)		
Employment			7754.0/−0.40	.69		7925.5/−0.10	.92
Unemployed	98 (37.5)	67.5 (17.1)			25.3 (100.8)		
Employed	163 (62.5)	67.7 (13.6)			27.2 (65.9)		
Perceived economic status			1.92	.38		2.18	.34
Low	145 (55.6)	67.4 (14.9)			27.2 (101.7)		
Middle	107 (41.0)	68.6 (13.4)			27.3 (56.0)		
High	9 (3.4)	62.5 (31.9)			23.0 (62.6)		
HIV care stage			0.39	.82		1.22	.54
Diagnosis to starting medication	10 (3.8)	65.6 (16.0)			10.7 (104.3)		
Starting medication to familiarization with medication	14 (5.4)	71.8 (25.4)			50.3 (98.6)		
Familiarization with medication to sustaining health management	237 (90.8)	67.8 (14.5)			27.3 (75.5)		
Self-help group participation			6014.0/−2.49	.01		6412.5/−1.79	.07
Yes	84 (32.2)	70.1 (13.1)			16.3 (90.9)		
No	177 (67.8)	65.8 (16.0)			30.1 (72.9)		

a U/Z: Mann-Whitney *U* test.

b H: Kruskal-Wallis test.

## Discussion

### Principal Results

mHealth interventions require careful consideration of user engagement levels, as this factor significantly influences both feasibility and clinical outcomes. Despite the expanding development of mHealth interventions, comprehensive engagement assessments remain limited in the existing literature. In this study, 2 complementary engagement indicators—engagement index and engagement time—were examined, and it was determined whether app engagement varies by participant characteristics. The results showed a median engagement index score of 67.6% and median engagement time of 27.2 minutes during the 28-day study period. Engagement differences were observed in only 1 area—the engagement index varied by self-help group participation—with no other characteristic-based variations identified. mHealth apps for people living with HIV are increasingly being developed across hospital outpatient clinics and community settings. Effective app development requires comprehensive evaluation plans that incorporate both objective indicators (such as login data) and subjective measures (such as patient experience) to thoroughly assess intervention effectiveness.

While mHealth targeting people living with HIV can deliver effective interventions through inclusive universal design principles, targeted facilitation strategies are needed for individuals with limited information access owing to lack of self-help group participation experience.

### Active User Trends

During the intervention period, the number of daily active users remained above 90, with the proportion of active users among all participants ranging between 34.5% and 54.4% by date. Considering that only 26% of people living with HIV are receptive to digital health care [17], and that the proportion of active users for diabetes self-management apps ranges from 1% to 38% per week during intervention periods [18], the level of daily active users in this study was notably high. This app was developed to reflect the results of health care service needs assessment for people living with HIV [19], and efforts were made to specifically provide the information they needed. Meanwhile, the app featured a medication recording function, which users evaluated as useful. It is considered that the daily user count remained relatively high owing to the nature of app usage, requiring daily logins to record medication intake. Furthermore, mileage points were awarded for using various features such as medication recording, Q&A, and commenting. Given that users have been reported to be 4 times more likely to maintain engagement when involved in a specific clinical condition and 20 times more likely to sustain participation when expecting a reward [20], it is reasonable to conclude that providing rewards for app usage contributed to increasing engagement [21,22].

### Derivation and Interpretation of the Engagement Index

The engagement index was modified to fit this study. The click-depth index was defined as the ratio of sessions with 4 or more page views among total sessions, applying a stricter criterion compared with previous studies that used sessions with 2 or more page views [23]. It has been reported that the engagement index can be designed to match study characteristics [12]. In this study, considering the unique characteristics that events such as “entering the home tab screen” necessarily occur to access the app, the aim was to identify sessions where actual health management activities were performed on the app. The engagement index provides objective and subjective measurements of usability through factors including login frequency, content usage volume, interaction frequency, and user experience. However, as it cannot track the time users spend on the app, engagement time was used as a complementary metric. These 2 indicators, while showing a statistically significant positive correlation, can be used complementarily as they capture distinct aspects of engagement. Moreover, the positive correlation between engagement time and engagement index suggests that securing content that enables users to spend sufficient time on the app may contribute to sustained engagement.

The average and median engagement index scores were 64.3% and 67.6%, respectively, significantly higher than in other studies using the same index to analyze app engagement, for example, the Growing Healthy study (30.0%), providing information on infant feeding from birth to 9 months [23], and the Milk Man study (29.7%), designed to engage fathers with breastfeeding and parenting information [24]. Given that the participants were people living with HIV, it can be considered that they had higher sustained app usage needs than other users with temporary needs, such as parents caring for newborns. Through preliminary needs assessment and pilot testing [19], strict privacy protection measures were implemented. Focusing on providing reliable health care information while incorporating design elements proved to be a positive factor in involving individuals who had previously been reluctant to actively participate in community engagement and information seeking. Furthermore, although not intended during the app design phase, the app’s characteristic requirement of daily medication recording significantly increased the values of the recency and loyalty subindices, which reflect “how frequently users access the app.” As noted in the study by White et al [24], it would be more appropriate to interpret the engagement index as an indicator reflecting app users’ behaviors rather than as a comparative measure of how well participants used the app.

### Factors Influencing Engagement

No differences in engagement were found based on other characteristics except self-help group participation, indicating that there are few people living with HIV subgroups requiring special approaches for app usage. This demonstrates that the app represents a health care service that is universally accessible without barriers, unaffected by factors such as age, marital status, or socioeconomic status. While targeting specific vulnerable groups based on population characteristics can be a strategic approach, this app provides valuable insights for developing universal mHealth interventions that are inclusive and accessible to all users.

The engagement index was higher among participants with self-help group participation. This is perhaps an expected result, as these are individuals who typically have a high interest in health management and actively seek information. Self-help groups serve as an effective and equitable platform for delivering health interventions, and conducting projects through self-help groups can increase trust in the research team while enhancing intervention participation through the sharing of useful information about app usage with one another [25]. Previous research has demonstrated that diabetes self-management interventions using self-help resources are effective in improving health outcomes [26]. In addition, the support of health care professionals, caregivers, and peer patients in the rehabilitation of those who have undergone arthroplasty can be a strategy to promote patient engagement in mobile technology-based interventions [27]. While this demonstrates that self-help groups can facilitate intervention implementation, it also suggests that efforts are needed to ensure that those who do not take part in self-help groups are not marginalized in obtaining app-related information. Researchers can enhance equity by including elements that promote communication among users or by sharing information obtained from users.

Previous studies have shown that higher age and higher educational levels are associated with higher levels of engagement with digital health or mHealth interventions [6,28]. In this study, while participants in their 40s showed notably higher median engagement time than those in other age groups and participants with college-level education or above had a higher engagement index than those with high school education or below, these differences were not statistically significant. The higher level of mHealth engagement among older adults is commonly attributed to their increased awareness of health importance, leading them to dedicate more time to health management [29]. In this study, while there was no significant difference in engagement index between age groups, the difference in engagement time suggests that older age groups may have required longer usage times because of lower familiarity with apps. While millennials focus on content for 12 seconds and prefer long-form content such as videos or podcasts, Gen Z focuses for only 8 seconds and prefers short and fast content such as Snapchat or Instagram Stories, which may reflect the shorter attention spans of younger generations [30]. This interpretation is supported by our analysis of usage patterns, which showed no age-related differences in content preferences. Furthermore, previous studies have reported that individuals with higher educational levels may demonstrate higher mHealth engagement owing to their superior digital health literacy, more positive attitudes toward technology, and enhanced ability to leverage social networks and support systems [31-33]. In our study, while the median engagement time was comparable between educational groups, the higher education group showed a higher median engagement index, which can be interpreted within this same framework.

Meanwhile, the differences according to age or educational level were not statistically significant. As confirmed in the distribution of engagement index and engagement time, it is worth considering the possibility that extreme values from some super users or participants with extremely low engagement rates may have diluted the differences between groups. Notably, the engagement index showed a relatively high level with a left-skewed distribution, while the engagement time exhibited a right-skewed distribution with significant asymmetry. Therefore, future research could benefit from qualitative approaches or latent profile analysis to closely examine the characteristics of individuals with high or low engagement levels, which would provide meaningful insights for enhancing mHealth intervention design.

### Limitations

It must be acknowledged that the results reflect selection bias. While participants in other comparative studies were recruited from a single institution or community [23,24], the participants in this study were recruited from 13 hospitals, where counseling nurses and physicians who distributed research announcements may have primarily guided individuals with good coping abilities, and those who were already proactive in health management and information seeking were more likely to enroll. In other words, it warrants consideration that the common characteristics of enrolled participants may have led to an overestimation of the results. While this study is significant in the development of an integrated nursing intervention app for continuous health care management of people living with HIV and the verification of engagement through indicators, the correlation between intervention engagement levels and participants’ health outcomes was not examined. It is expected that future studies demonstrating this correlation will provide evidence that app intervention engagement can have positive effects on health outcomes in people living with HIV.

### Conclusions

Engagement is a valuable metric for predicting intervention effectiveness. With a substantial sample size and institutional representation, this study provides a comprehensive assessment of engagement with mHealth intervention among people living with HIV. Key findings highlight the need for user-centric app development incorporating rewards, privacy protection, and tailored information, as well as targeted support strategies for groups with lower engagement levels, such as those with no self-help group participation.

## Abbreviations

ESSC: Excellent Self-Supervised HIV Care
Health-ITUES: Health Information Technology Usability Evaluation Scale
mHealth: mobile health
Q&A: question and answer
